# Contribution de la dérivation AVR dans le diagnostic de la cardiopathie ischémique: à propos d’un cas à l’Institut de Cardiologie d’Abidjan (Côte d’Ivoire)

**DOI:** 10.11604/pamj.2022.42.311.20166

**Published:** 2022-08-25

**Authors:** Loa Ambroise Gnaba, Kassi Anicet Adoubi, Kouakou Florent Diby, Isabelle Kouamé, Pinnin Evelyne Adjara Ouattara, Manga Diomandé, Mbe Matokoma Daniogo, Keumian Gabin Tro, Koudré Serge Armel Dakoi, Aka Roland N’Guetta

**Affiliations:** 1 Service des Maladies Cardiovasculaires et Thoraciques, Centre Hospitalier Universitaire Bouaké, Bouaké, Côte d´Ivoire,; 2 Université Alassane Ouattara, Bouaké, Côte d´Ivoire,; 3 Institut de Cardiologie d´Abidjan, Abidjan, Côte d´Ivoire,; 4 Université Felix Houphouët-Boigny, Cocody, Côte-d´Ivoire

**Keywords:** Dérivation aVR, sus-décalage de ST, ondes Q de nécrose, Cote d’Ivoire, cas clinique, aVR lead, multivessel coronary artery disease, case report

## Abstract

Des anomalies dans la dérivation aVR fourniraient des informations utiles sur le risque de maladies coronariennes. Ce présent cas clinique en est une illustration. En effet, il s´agit d´un patient de 60 ans, tabagique actif et diabétique ancien type 2 qui a présenté une douleur thoracique d´allure angineuse avec une épreuve d´effort positive. L'électrocardiogramme initial a montré un discret sus-décalage du segment ST et une onde Q de nécrose en aVR avec des signes en miroir en territoire inférieur. Une cardiopathie ischémie à fraction d´éjection ventriculaire altérée a été objectivée. La coronarographie diagnostique a objectivé une atteinte pluritronculaire coronaire. En définitive, la dérivation aVR fournit des informations cliniques précieuses et plaide en faveur d´une attention particulière à cette dérivation souvent oubliée.

## Abstract (en)

Abnormalities in the aVR lead would provide useful information on the risk of coronary heart disease. This clinical case is an illustration. Indeed, this is a 60-year-old patient, an active smoker and a former type 2 diabetic who presented with angina-like chest pain with a positive stress test. The initial electrocardiogram showed a discreet elevation of the ST segment and an aVR necrosis Q wave with mirror signs in the inferior territory. An ischemic heart disease with altered ventricular ejection fraction was objectified. The diagnostic coronary angiography objectified a multi-vessel coronary lesion. Ultimately, the aVR lead provides valuable clinical information and argues for special attention to this often forgotten lead.

## Introduction

L'électrocardiogramme 12 dérivations est un outil diagnostique essentiel en cardiologie. Contrairement aux onze autres dérivations, la dérivation aVR a longtemps été négligée jusqu'à ces dernières années [1]. Des études récentes ont montré qu'une analyse minutieuse de cette dérivation fournit de nombreuses informations pouvant être regroupées en trois indications [2,3]. La première indication porte sur le bilan de la cardiopathie ischémique. En effet, cette dérivation permet de prédire de l'artère coupable et d´estimer l´extension des lésions afin d´en évaluer le pronostic. La deuxième indication est relative au bilan des anomalies du rythme cardiaque et de la conduction. Il s´agit du diagnostic des troubles de la conduction intraventriculaire (hémibloc antérieur gauche, bloc de branche droit), de la prédiction de l'origine des arythmies ventriculaire et supraventriculaire et de la prédiction de l'arythmie dans la cardiomyopathie hypertrophique. Dans la troisième indication relative aux conditions diverses, la dérivation aVR aide au diagnostic de plusieurs affections cardiovasculaires dont la péricardite aiguë, la cardiomyopathie hypertrophique et l´embolie pulmonaire (Tableau 1) [2,4]. Sur l´ensemble des indications suscitées, la présente étude porte sur une illustration dans laquelle cette dérivation aVR fournit des informations en faveur du diagnostic de la cardiopathie ischémique avec une atteinte coronarienne pluritronculaire.

**Tableau 1:** situations où la dérivation aVR peut être utile

**CARDIOPATHIE ISCHEMIQUE**
Prédiction de l'artère coupable dans le syndrome coronarien aigu
Estimation de l'extension du syndrome coronarien aigu
Évaluation du pronostic dans le syndrome coronarien aigu
Évaluation de l'électrocardiographie d'effort
**ANOAMLIES DU RYTHME ET DE LA CONDUCTION**
Diagnostic d'hémibloc antérieur gauche, bloc de branche droit complet ou incomplet
Prédiction de l'origine de l'arythmie ventriculaire
Prédiction de l'origine de l'arythmie supraventriculaire
Prédiction de l'arythmie dans la cardiomyopathie hypertrophique ou l'intoxication aux antidépresseurs tricycliques
**CONDITIONS DIVERSES**
Diagnostic de mauvais positionnement des électrodes
Axe du Coeur
Hypertrophie ventriculaire droite
Péricardite aiguë
Cardiomyopathie hypertrophique
Cardiomyopathie post-partum
Embolie pulmonaire

## Patient et observation

**Informations relatives aux patients**: patient de 60 ans, aux antécédents de tabagisme actif en raison de 16 paquets, de diabète type 2 ancien (environ 06 ans) sous insulinothérapie qui a été adressé au service d´Hémodynamique de l´Institut de Cardiologie d´Abidjan (ICA) pour une coronarographie diagnostique d´une douleur thoracique d´allure angineuse. On notait dans l´histoire des épisodes de douleurs thoraciques à l´effort avec une épreuve d´effort positive ayant fait évoquer un angor stable. On notait également des claudications intermittentes aux membres inferieurs depuis environ deux ans. Il n´y avait pas de notions de dyspnée, de palpitions ni de malaises.

**Résultats cliniques**: l'examen physique à l´entrée a révélé un sujet en bon état général, apyrétique avec une fréquence cardiaque à 65/minute, une fréquence respiratoire à 19/minute et une pression artérielle de 120/60 mmHg. Il n´y avait pas de notions de douleur thoracique active, de dyspnée, ni de signes périphériques d´insuffisance cardiaque. Les pouls pédieux étaient diminués d´intensité à la palpation. L´auscultation cardiaque et des axes artériels était sans particularité. L´examen des autres appareils notamment neurologique, pleuropulmonaire et digestif était sans particularité.

**Chronologie**: devant l´examen clinique satisfaisant, le patient a été proposé à la coronarographie. Un bilan pré-thérapeutique a été demandé à cet effet.

**Figure 1:**
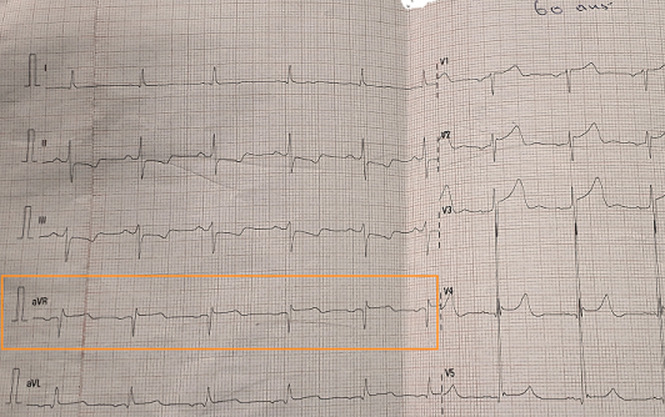
image électrocardiographique montrant un sus-décalage du segment ST et une onde Q de nécrose en aVR et des images en miroir en inférieur (encadré rouge)

**Démarche diagnostique**: l'électrocardiogramme initial réalisé a montré un sus-décalage persistant du segment ST de 0,1 mV, une onde Q de nécrose de grande amplitude (6mm) et de durée égale à 0,02 seconde en aVR uniquement et des images en miroir en territoire inférieur (Figure 1). L´échocardiographie a permis d´objectiver une dysfonction systolique modérée (fraction d'éjection à 50%) et une akinésie de la paroi septo-apicale du ventricule gauche. Les résultats de laboratoire pertinents ont montré un taux de Troponine I normal (Troponine I=0,025ng/ml). Le reste du bilan à savoir la fonction rénale (l´urée, la créatininémie) et le bilan d´hémostase (taux de Prothombine, le temps de céphaline kaolin) était normal.

**Figure 2:**
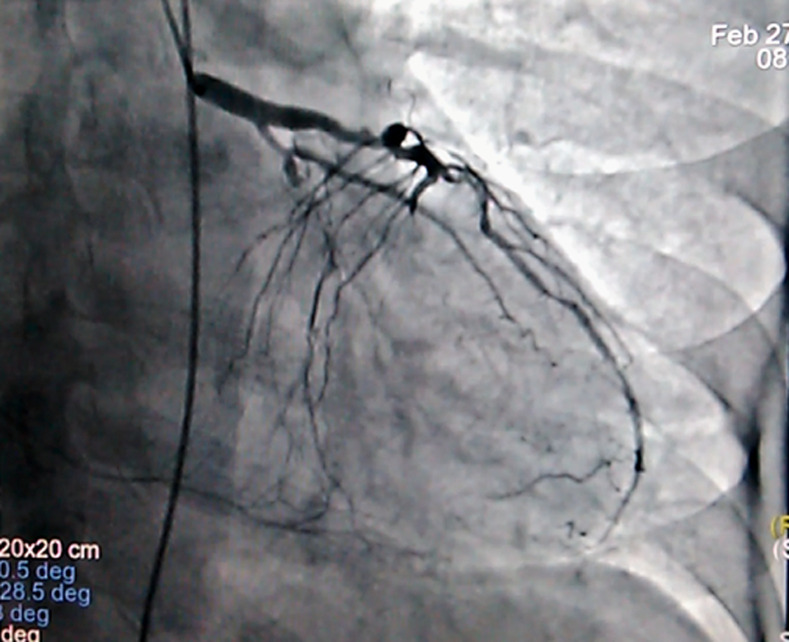
image de coronarographie montrant en incidence OAD caudale une lésion serrée à 70-80% de l´IVA moyenne, une occlusion homocontrolatéralisée de l´IVA apicale et de la CX moyenne et une occlusion controlatéralisée de la CD proximale (IVA: artère inter-ventriculaire antérieure, CX: artère circonflexe, CD: artère coronaire droite, OAD: oblique antérieur droite)

**Intervention thérapeutique**: la coronarographie a été réalisée le lendemain. Après une anesthésie du point de ponction (Xylocaïne 2%), l´artère radiale a été ponctionnée selon la technique de Seldinger. Un désilet qui a la particularité d´avoir une valve étanche au reflux sanguin a été mis en place à travers lequel sont passées les sondes de coronarographie. La montée des sondes coronaires s´est faite sous scopie avec prudence, sans résistance. Les résultats montraient une atteinte pluri tronculaire coronaire attestée par une lésion serrée à 70-80% de l´artère interventriculaire antérieure moyenne, une occlusion homocontrolatéralisée des artères interventriculaire apicale et circonflexe moyenne et une occlusion controlatéralisée de la coronaire droite proximale (Figure 2). Devant l´atteinte pluri tronculaire des lésions il a été indiqué un pontage aorto-coronarien. Le patient a été proposé à la chirurgie vasculaire.

**Suivi et résultats des interventions thérapeutiques**: en fin de procédure, le patient avait bénéficié d´un traitement classique à base de Statine (Atorvastatine), de double antiagrégants plaquettaires (Acide acétylsalicylique, Clopidogrel) de Beta bloquants (Bisoprolol), d´Inhibiteur de l´Enzyme de Conversion (Ramipril) et de sensibilisation à l´arrêt du tabac. En attente de sa prise en charge spécialisée, un suivi régulier tous les six mois a été proposé.

**Perspectives du patient**: avant son admission en salle de cathétérisme, le patient a bénéficié d´une explication sur la procédure avec un document à l´appui pour pouvoir obtenir son avis. Il lui a été signifié les anomalies objectivées et la suite de la prise en charge chirurgicale.

**Consentement éclairé**: un consentement éclairé écrit, daté et signé a été obtenu du patient ayant permis la réalisation de ladite exploration.

## Discussion

Le présent cas clinique présente des anomalies de la dérivation aVR dans un contexte de syndrome douloureux thoracique chez un sujet à haut risque avec atteinte pluri tronculaire coronaire compliquée d´une cardiopathie ischémique. C´est une contribution à l´étude de la dérivation aVR dans l´interprétation d´un électrocardiogramme (ECG) et à la problématique de l´implication clinique du sus-décalage persistant du segment ST et de l´onde Q de nécrose. Concernant le sus décalage du segment ST, Kosuge *et al*. [5] l´ont aussi objectivé dans leur série chez des patients avec des atteintes du tronc commun (TC) et/ou tritronculaires (TTC) avec une sensibilité de 78% et 86% de spécificité. De même, Yamaji *et al*. ont montré la contribution de cette dérivation dans la cardiopathie ischémique [4]. Ils ont en effet trouvé qu'un sus décalage du segment ST plus important en aVR qu'en V1 en termes d´amplitude est sensible à 81 % et spécifique à 80 % de l´atteinte de la coronaire gauche [4-7]. L´absence d´anomalies dans d´autres territoires à l´ECG notamment dans les dérivations précordiales dans notre cas trouve son explication dans plusieurs travaux. D´abord celui de Yamaji *et al*.qui ont montré que l'altération simultanée du flux sanguin à la fois dans les artères circonflexe gauche et interventriculaire antérieure pourrait rendre le vecteur du segment ST plus perpendiculaire à la dérivation V1, entraînant une moindre élévation du segment ST dans ladite dérivation [4]. Ensuite d´autres travaux ont montré l'implication du septum basal. En effet, la dérivation aVR faisant face au septum basal plus que toute autre dérivation, qui a une double perfusion à la fois de l'artère coronaire droite et de l'artère interventriculaire antérieure, peut entraîner un sus-décalage du segment ST dans la dérivation aVR. La perfusion de l'artère interventriculaire antérieure gauche passant par la première branche septale, l'occlusion de la première branche septale ou de l'artère interventriculaire antérieure gauche en amont de la première branche septale peut également provoquer un sus-décalage du segment ST en aVR. Ainsi, en raison de la double perfusion du septum basal, le sus décalage du segment ST en aVR dans le syndrome coronarien aigu (SCA) devrait suggérer une maladie multitronculaire ou une maladie du tronc commun [4,8,9].

En conséquence, les lésions coronariennes sévères obtenues dans notre cas et celles de la littérature décrites plus haut permettent de conclure avec Uzun *et al*. [2] que le sus-décalage du segment ST dans la dérivation aVR pourrait prédire de la sévérité des atteintes dans la cardiopathie ischémique. Par ailleurs, le présent cas clinique présente un sus décalage du segment ST en aVR qui contraste avec une troponine I négative. Ce contraste s´expliquerait dans le travail d´Assali *et al*. [10] qui montre que le sus-décalage du segment ST dans la dérivation aVR est l'inversion des dérivations V5 et V6. Toute situation entraînant un sous décalage du segment ST en V5 et V6 pourrait entraîner un sus-décalage du segment ST en aVR. C´est pourquoi, l'ischémie sous-endocardique antérolatérale, qui peut provoquer un sus-décalage du segment ST dans la dérivation aVR entraine un sous-décalage du segment ST dans les dérivations V5 et V6 [10,11]. Kosuge *et al*. [5] faisaient la même remarque dans leur travail portant sur le SCA sans sus décalage du segment (SCA ST-), où ils retrouvaient un sus décalage du segment ST ≥ 0,05 mV en territoire aVR. En conclusion, un sus décalage de ST en aVR ne traduit pas nécessairement un SCA avec sus décalage du segment (SCA ST+). S´agissant de l´onde Q de nécrose, elle apparaît dès la 6^e^ heure dans un SCA et traduit la nécrose transmurale du myocarde pouvant se compliquer d´un trouble de la cinétique ventriculaire gauche comme retrouvé dans notre cas. Cette onde Q en aVR a été objectivé également par Wagner *et al*. [11]. Ils ont retrouvé dans le groupe des patients avec une onde Q de nécrose, une prévalence élevée d´une atteinte de l´IVA, une hypokinésie des régions apicale et inférieure avec une fraction d´éjection du ventriculaire gauche altérée comparativement au groupe contrôle. Ces résultats de la littérature sont quasi superposables à notre cas.

## Conclusion

La dérivation aVR a contribué au diagnostic de la cardiopathie ischémique par atteinte coronarienne sévère chez un sujet à haut risque cardiovasculaire. De ce fait, aucune dérivation ne doit être négligée lors de l'interprétation d´un Electrocardiogramme.
